# Low-cost nano biochar: a sustainable approach for drought stress mitigation in faba bean (*Vicia faba* L.)

**DOI:** 10.3389/fpls.2024.1438893

**Published:** 2024-11-12

**Authors:** Heba Ibrahim Abd El-Moaty, Ali El-Dissouky, Amel F. Elhusseiny, Kareem M. Farag, Rasha Abu-Khudir, Mayyadah Abdullah Alkuwayti, Najla K. Al Abdulsalam, Salwa M. Abdel Rahman

**Affiliations:** ^1^ Department of Biological Sciences, College of Science, King Faisal University, Al-Ahsa, Saudi Arabia; ^2^ Medicinal and Aromatic Plants Department, Desert Research Center El-Mataria, Cairo, Egypt; ^3^ Chemistry Department, Faculty of Science, Alexandria University, Alexandria, Egypt; ^4^ Department of Chemistry, College of Science, King Faisal University, Al-Ahsa, Saudi Arabia; ^5^ Biochemistry Division, Department of Chemistry, Faculty of Science, Tanta University, Tanta, Egypt; ^6^ Botany and Microbiology Department, Faculty of Science, Alexandria University, Alexandria, Egypt

**Keywords:** broad bean, nanoparticles, green chemistry, biochar, drought

## Abstract

This study tends to reach some objectives of the sustainable development goals, which call for responsible consumption and production and climate action. Long-term global food security is affected by drought and the optimal use of water in agricultural areas under climate change scenarios. Our approach aims to amend soil for cultivation under drought stress and improve plant growth to contribute to food security. In this context, a biochar was prepared from peanut shell and thoroughly examined as a soil enhancer for broad bean cultivation during drought stress. The produced biochar exhibited 0.307 g cm^−3^ bulk density, 9.6 cmol kg^−1^ cation exchange capacity, −15.5 mV zeta potential, and an average diameter of 21.86 nm. Surprisingly, the application of biochar increased soil water holding capacity and organic matter by 66% and 220%, respectively. Moreover, its application under drought improved plant growth as indicated by stem height, leaf area index, pod number/plant, pod weight, protein level, chlorophyll content, nutrient levels in leaves, and reduced lipid peroxidation and electrolyte leakage. The principal component and factorial analysis of the current study demonstrated correlations between the physiological response of faba bean plants and soil physiochemical parameters after the application of peanut shell-derived biochar. This study presents promising nano biochar that could be an effective sustainable practice for disposing residual materials.

## 1 Introduction

Crop yields are being negatively impacted by climate change and will continue to do so. On average, it is projected that crop yields have decreased by 70% since 1982 as a result of climate change, with very few agricultural areas being undisturbed (Food and Agriculture Organization of the United Nations). Drought has been known as the primary cause of the decline in agricultural output, which is predicted to increase in the face of global climate change. Globally, more than 34% of the loss in crop and livestock production is due to drought stress. Drought causes a series of physiological, biochemical, and molecular changes in plants. It negatively affects plants through disrupting nutrient uptake, damaging organelle structures, inducing the decomposition of chlorophyll, inhibiting photosynthetic activity, and suppressing leaf growth (Farooq et al., 2008; Osakabe et al., 2014; Fahad et al., 2017). Under drought stress, reactive oxygen species (ROS) are increasingly produced, causing oxidative stress (OS) and accelerating the aging process (Rizwan et al., 2015). Consequently, overcoming drought is a difficult issue for achieving global food security. To counteract drought stress, organic amendments including composts, digestates, manures, and biochar are added to the soil to increase its water holding capacity (WHC). Many strategies have been used to alleviate drought stress in plants such as exogenous application of β-aminobutyric acid (Abid et al., 2020), gibberellic acid and bee honey boosts (Rady et al., 2021), as well as plant growth-promoting bacteria (PGPB) (Harkhani and Sharma, 2024), melatonin treatment (Amiri et al., 2024), seed priming with selenium (Se) (Raza et al., 2024), and biochar amendments (Gullap et al., 2024).

Biochar is a carbon-rich material produced by the thermal decomposition of biomass under limited oxygen conditions (Ali et al., 2017; Kumar et al., 2020). Biochar can be produced from different types of biomass, including agricultural wastes, wood residues, food wastes, animal manure, and sludge (Wijitkosum, 2022). Previous studies have reported that biochar amendments improve the physiochemical characteristics of soil such as nutrient availability, WHC, pH, and cation exchange capacity (CEC) as well as the mitigation of climate change through carbon sequestration and reduction of greenhouse gases (Roberts et al., 2010; Rafael et al., 2019; Rollon et al., 2020). In addition, biochar is applied in the remediation of contaminated soil and wastewater treatment due to its ability to absorb undesirable contaminants (Sattar et al., 2019; Yang et al., 2019; Zhu et al., 2020; Afzal et al., 2024). It has been shown that the structure of biochar exhibits a notable degree of stability when evaluated from the point of view of microbial and chemical degradation. This stability sets biochar apart from other organic compounds and can offer long-term advantages to soil (Agegnehu et al., 2015). Previous research has demonstrated that biochar application improved plant growth and yield of several plants under drought stress, including tomato (Akhtar et al., 2014), okra (Batool et al., 2015), maize (Haider et al., 2015), grape (Genesio et al., 2015), rapeseed (Lalay et al., 2022), and soybean (Gullap et al., 2024). Applying biochar has been shown to improve nutrient intake, soil microbial activity, photosynthetic rate (Diamadopoulos, 2019), and the plant’s defense mechanisms against drought by increasing the activity of antioxidant enzymes. This reduces the damage that drought causes to the photosynthetic machinery (Lyu et al., 2016). The vital role of biochar in improving soil WHC is a key factor in enhancing plant growth and yield during drought stress (Foster et al., 2016; Licht and Smith, 2017). Notably, because of its nano size, nano biochar is applied to increase crop yield and improve soil fertility (Shafiq et al., 2023). Previous studies have shown that applying nano biochar increased plant growth and yield in a number of drought stressed plants such as broccoli (Azadifar et al., 2023), wheat (Raza et al., 2023), and spinach (Rasheed et al., 2024).

Faba bean (*Vicia faba* L.) is a major food in the diets of inhabitants of the Middle East, the Mediterranean region, and China because of the high nutritional value of its seeds (Chaieb et al., 2011; De Cillis et al., 2019). It is a significant source of protein-rich food, and its nutritional value is superior to that of peas or other grain legumes (Crépon et al., 2010). It has a crucial role in improving the fertility of the soil by its rotation cultivation with cereal crops due to its ability to fix atmospheric nitrogen, thereby rendering them excellent colonizers of low-N environments (Abid et al., 2020). It is one of the most significant food legume crops cultivated in Egypt. For the year 2020, FAO statistics indicated that the areas cultivated with faba beans in Egypt decreased from 110,100 to 32,532 ha during the past 50 years and the national self-sufficiency rate also decreased from 70% to 26.9%. The reduction in faba bean yield could be related to changing environmental conditions (Quarshie et al., 2021; Abo-Hegazy and Darwish, 2022). According to Abid et al (Abid et al., 2020), drought is one of the main environmental factors that can significantly restrict the growth and productivity of faba bean plants. Martínez et al. (2007) reported that faba bean requires more water and is more susceptible to drought compared to other legume crops. Accordingly, drought stress can cause a significant decline in faba bean productivity, thus impacting agriculture and sustainable development in Egypt.

The current study was designed to evaluate the effect of application of the derived biochar as a soil amendment on growth, photosynthetic pigments, mineral nutrition, protein pattern, lipid peroxidation, and electrolyte leakage (EL) on faba bean plants under drought stress. Additionally, this study aimed to identify an alternative method for disposing peanut shells considered agricultural waste, which copes with the sustainable development goals declared by the United Nations (Farooq et al., 2008; Ali et al., 2017; Abid et al., 2020; Kumar et al., 2020). To the best of our knowledge, it is the first time to prepare biochar in the nano scale from peanut shell.

## 2 Materials and methods

### 2.1 Synthesis of nano biochar

Peanut was purchased from the local market. Peanut shells were washed with water in order to remove dust and mud impurities and then air-dried. Afterwards, the shells were ground well using a mechanical blender. A certain amount of the peanut shells was put in a closed reactor system and then heated to 450°C for 1.5 h. The obtained product is biochar (Jiang et al., 2022).

### 2.2 Physical and chemical characterization of the synthesized biochar

#### 2.2.1 Production of nano biochar

The given equation was applied to estimate the production rate:


Production rate(%)=(wt of biochar/wt of peanut shell)×100

#### 2.2.2 Electrical conductivity and pH

The electrical conductivity (EC) measurement was performed using a conductometer (WTW-LF-91, England), and the pH was recorded by means of a glass electrode pH meter (WTW model 512).

#### 2.2.3 The bulk and trapped density

The bulk density (BD) for peanut shell and its produced biochar was calculated according to Wang and Kinsella’s method (Wang and Kinsella, 1976), whereas a graduated cylinder was filled with a known quantity of powdered samples that were dried and weighed. The cylinder was then tapped for a few minutes to compact the sample, the volume of the sample was verified in milliliters, and BD was calculated using the given equation:


$$\begin{array}{c} \text{Bulk density }\left({\text{g mL}}^{-\;1}\right)\\ =\text{weight of dry material }\left(\text{g}\right)/\text{volume of dry material }\left(\text{mL}\right) \end{array}$$

The tapped density was estimated in the same way, but by tapping the cylinder containing the powder for a fixed number, then applying the given equation:


$$\begin{array}{c} {\text{Tapped density g mL}}^{-1}\\ =\text{weight of powder }\left(\text{g}\right)/\text{the tapped volume of powder }\left(\text{mL}\right) \end{array}$$

#### 2.2.4 Surface area measurement

Sears’ method (Sears, 1956) was applied to assess the surface area of the biochar rapidly. Accordingly, 1.5 g of the biochar was agitated in 100 mL of diluted HCl at pH 3. Sodium chloride (NaCl, 30 g) was added and stirred, and the volume was completed to 150 mL using deionized water. Titration was carried out with 0.1 N NaOH to increase the pH up to 9 and the volume (*V*) is verified. The surface area was estimated using the equation:


$$\begin{array}{c} \text{S}\left({\text{m}}^{2}{\text{g}}^{-1}\right)=32\text{V}–25,\\ \text{ Where V}=v\text{olume of }0.1\text{ N NaOH used to elevate the biochar pH up to }9 \end{array}$$

#### 2.2.5 Cation exchange capacity

To deduce the CEC of the biochar, the ammonium acetate displacement method (Gaskin et al., 2008) was carried out. Deionized water (20 mL) was used to leach 0.20 g of biochar five times. The leachates were then collected together, then the biochar was again leached with 20 mL of 1.0 M sodium acetate (pH 7) five times and collected together as exchangeable base cations (potassium, calcium, and magnesium). Ethanol was used to wash the biochar samples from any excessive sodium. Finally, 20 mL of 1 M ammonium acetate (pH 7) was applied to displace the sodium ions on the synthesized biochar. The CEC of the biochar was investigated from the sodium displaced by ammonium ion. The contents of sodium ions in the solution were detected by JENWAY PFPF7 flame photometry.

#### 2.2.6 FT-IR spectra

FT-IR spectra of the peanut shell and its biochar were recorded using a Perkin-Elmer FT-IR 1650 Spectrophotometer ranging from 4,000 to 500 cm^−1^ at 25°C from KBr pellets of thickness 3 mm.

#### 2.2.7 Zeta potential and size distribution

The biochar surface charges were determined by Zetasizer (Nano ZS Malvern).

#### 2.2.8 Surface morphology studies

The morphologies of nanoparticles were displayed by a JEOL JSM-IT200 Scanning Electron Microscope (SEM) (Tokyo, Japan) at the Electron Microscopy Unit of the Faculty of Science, Alexandria University, Alexandria, Egypt. Sonication of the sample in deionized water for 5 min was carried out, deposited onto carbon-coated copper mesh, and dried before examination. A SEM along with an energy dispersive x-ray spectroscope (EDX) were operated to examine the elemental composition of biochar.

### 2.3 Experimental conditions

The experiment was conducted at the greenhouse of the Faculty of Science, Alexandria University under a complete randomized design. Three seeds were planted in each pot on 15 November until the second half of March under the following environmental conditions: temperature ranged from 18 to 26°C, relative humidity ranged from 50% to 60%, and average daylight from 10 to 14 hours/day. The experimental design consisted of the following treatments: B0 = no biochar, B1 = 2% nano biochar (w/w), B2 = 4% nano biochar (w/w), where 80% pot WHC was treated as control (C), whereas 50% pot WHC was considered as drought stress (D).

### 2.4 Soil analysis

Samples of control soil and soil amendment with 2% or 4% nano biochar were spread over sheets of paper in the air until they are dry, thoroughly mixed, ground well, passed through a 2-mm sieve to remove gravel and debris, and then packed in plastic bags. The sieved soil samples were analyzed for some of their physical and chemical properties according to Blume et al (Blume et al., 1982). EC and soil pH were measured in a 1:5 soil water extract using an EC meter (WTW-LF-91, England) and a glass electrode pH meter (WTW model 512). Texture and particle size distribution (PSD) for surface samples were determined using a hydrometer and sieves (Klute, 1986). Organic matter content (OM %) was quantified by using the potassium dichromatic oxidation titration technique according to the Walkley and Black method (Walkley and Black, 1934). Soil water holding capacity (SWHC) was measured by the gravimetric method according to Klute (1986). Sodium adsorption ratio (SAR) was calculated according to the following formula as described by Lesch and Suarez (2009):


$$SAR=\frac{Na^{+}}{\sqrt{\frac{Ca^{2+}+Mg^{2+}}{2}}}$$

Nitrogen (N) was assessed by the Kjeldahl method. Bicarbonates (HCO_3_^−^) were appraised by the titration method. Phosphorus (P) was digested using perchloric and sulfuric acids and then was analyzed using the molybdenum antimony blue colorimetric method (Olsen and Dean, 1965). Available potassium (K), magnesium (Mg), calcium (Ca), and some micronutrients were determined by using a flame photometer and an atomic absorption spectrophotometer according to Allen et al (Allen et al., 1974). Total calcium carbonate content (CaCO_3_%) was determined using the pressure calcimeter method (Blume et al., 1982).

### 2.5 Growth and yield parameters

For each treatment, plant height was measured, and leaf area was estimated using the Milford et al. (1985) equation. Yield parameters, including pod number/plant (PN/P), grain number/pod (GN/P), and weight of 100 seeds, were recorded at the maturity stage. Results are presented as the mean of three replications in each treatment.

### 2.6 Determination of chlorophyll a and b and total chlorophyll

The photosynthetic pigments’ total chlorophyl (TCh), chlorophyll a (Cha), and chlorophyll b (Chb) were measured spectrophotometrically at 647 and 664 nm following the *N*,*N*-dimethylformamide (DMF) method as described by Inskeep and Bloom (1985). The following formula and extinction coefficients were utilized to determine the photosynthetic pigments:


$$\text{Cha}=12.70{\text{ A}}_{665}–2.79{\text{ A}}_{647}$$

$$\text{Chb}=20.70{\text{ A}}_{647}–4.62{\text{ A}}_{665}$$

$$\text{TCh}=17.90{\text{ A}}_{647}+80.08{\text{ A}}_{665}$$

The chlorophyll stability index (CSI %) was calculated according to the following formula as described by Sivasubramaniawn (1992):


(TCh content in stressed leaves/total Ch content in control leaves)×100

### 2.7 Protein assay and SDS-PAGE

Protein concentration was estimated as described by Bradford (1976) using bovine serum albumin (BSA) as the standard. Protein electrophoresis was carried out using Laemmli’s SDS-PAGE technique (Laemmli, 1970). The molecular weight of proteins was analyzed with gel image analysis software (TotalLab V.1.0.0.1).

### 2.8 Analysis of nutrient levels in leaves

Oven-dried broad leaves from different treatments were digested according to the methods of Jones and Case (1990). The Kjeldahl method was used to estimate the total nitrogen. Sulfur was examined according to the method of Okalebo et al (Okalebo et al., 2002). Mg, Ca, Cu, Fe, Mn, and Zn were measured using an atomic absorption spectrophotometer (Perkin-Elmer 100B) following Isaac (1980). A spectrophotometer (T80+, PG Instruments Limited, Leics, United Kingdom) was used to measure the amount of phosphorus using the vanadate–molybdate technique at 660 nm (Chapman and Pratt, 1962). A Perkin-Elmer Flame photometer was used to measure potassium and sodium (Blume et al., 1982).

### 2.9 Determination of lipid peroxidation

The level of lipid peroxidation was measured according to the thiobarbituric acid (TBA) test, which determines the malondialdehyde (MDA) content as the product of the lipid peroxidation reaction. MDA concentration was measured using a spectrophotometer and calculated by applying its extinction coefficient 155 mM^−1^ cm^−1^ (Heath and Packer, 1968).

### 2.10 Electrolyte leakage

Plant samples were immersed in deionized water and then placed in a water bath held at a constant temperature of 32°C. After 2 h, the initial EC of the medium (EC1) was recorded. The samples were then autoclaved at 120°C for 20 min, and after cooling, the final EC (EC2) was measured. The percentage of EL was calculated using the formula: EL% = (EC1/EC2) × 100 (Dionisio-Sese and Tobita, 1998).

### 2.11 Data analysis

Analysis of variance (ANOVA) was performed using IBM SPSS v.27.0 software to evaluate the interaction between drought and nano biochar application. The principal component analysis (PCA) was performed using Sigma Plot program V.15, correlograms were carried out using R software V.4.3.1 (Team, R.C, 2013), and factorial analysis was performed by using Design-Expert V. 13.

## 3 Results

### 3.1 Characterization of the prepared nano biochar

**Figure 1:**
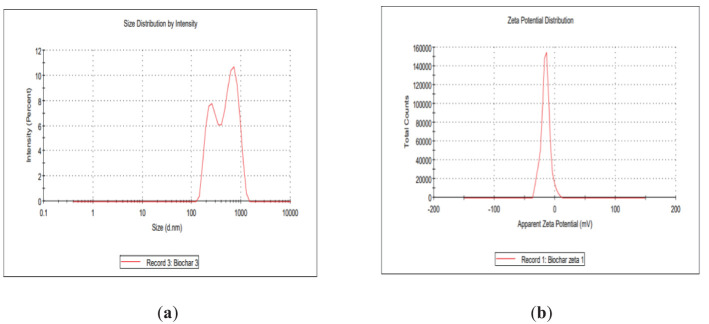
**(A)** Size distribution and **(B)** zeta potential analysis of the prepared nano biochar.

**Figure 2:**
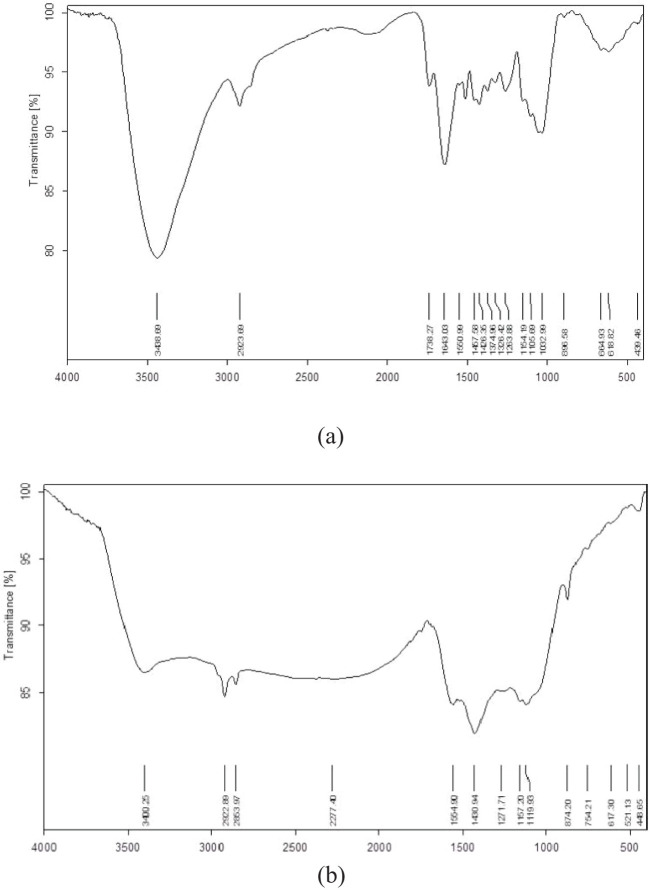
FTIR spectrum of **(A)** the peanut shell feedstock and **(B)** the prepared nano biochar.

The rate of nano biochar production was 32%. The EC of the prepared nano biochar was found to be 3.40 dS m^−1^. The measured pH value of the nano biochar was 7.4. The BDs of the peanut shells and the derived nano biochar were 0.212 and 0.307, whereas their trapped densities were 0.226 and 0.422, respectively. The values of the bulk and trapped density for peanut shells were less dense than their nano biochar. The observed experimental CEC value of the prepared nano biochar was 9.6 cmol kg^−1^. The surface area of the prepared nano biochar was 208.2 m^2^ g^−1^. The PSD of fine, medium, and coarse nano biochar was measured (**Figure 1A**) and a Z average size of 606.2 nm was reported, whereas the zeta potential value was −15.5 mV (**Figure 1B**), suggesting negative charges on the nano biochar. The analysis of the FTIR spectrum of the peanut shell and nano biochar is presented in **Figure 2**, **Table 1**.

**Table 1:** FTIR of peanut shell feedstock and its nano biochar.

Compound	ʋ (cm^−1^)
Peanut shell	3,438, 2,923, 1,738, 1,643, 1,550, 1,457, 1,426, 1,374, 1,326, 1,263, 1,154, 1,105, 1,032, 896, 664, 618, 439
Nano biochar	3,400, 2,922, 2,853, 2,277, 1,554, 1,430, 1,271, 1,157, 1,119, 874, 754, 617, 521, 448

The analysis of FTIR spectrum of the peanut shell and nano biochar is presented in **Table 1**, **Figure 2**.

**Figure 3:**
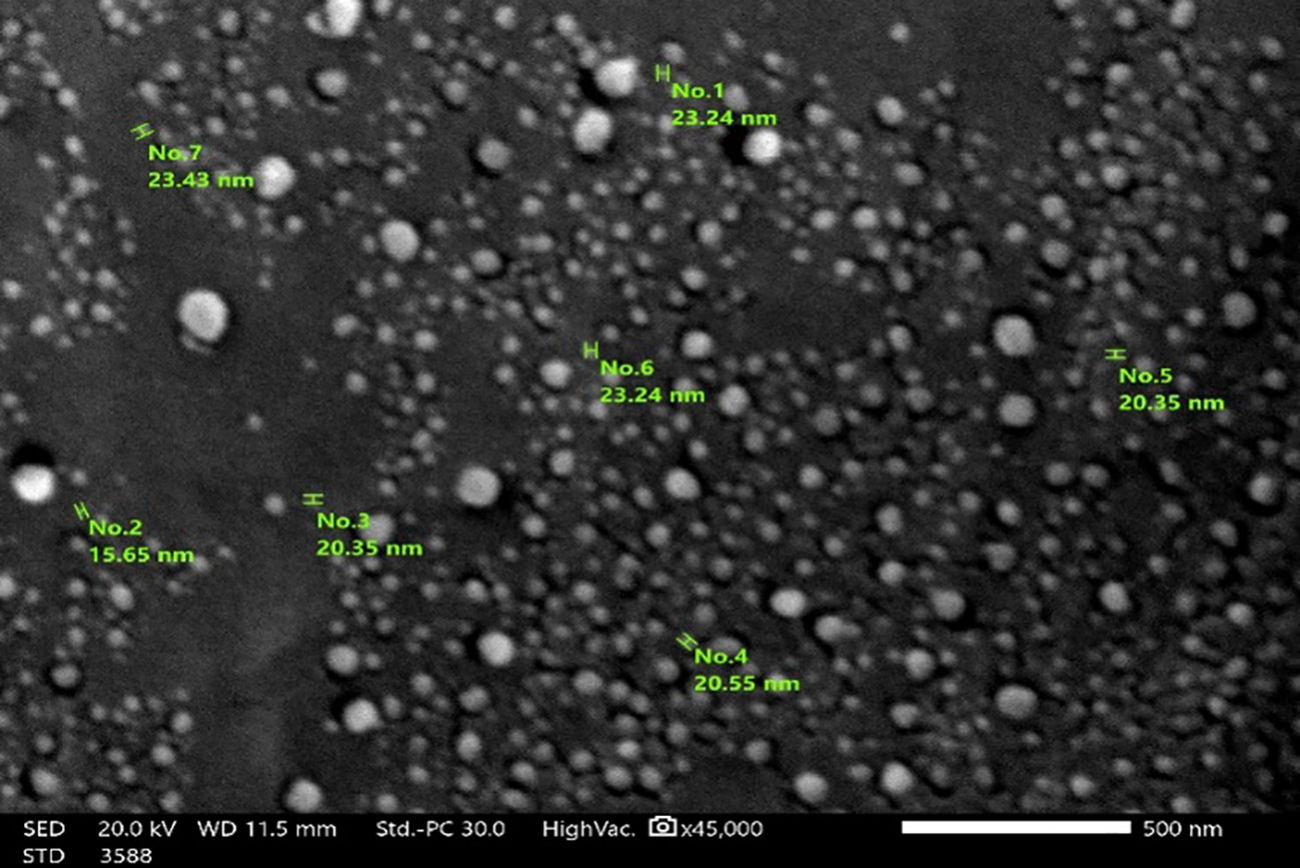
Scanning electron microscope (SEM) micrograph of the prepared biochar.

The particles’ average diameter of the prepared biochar was investigated from SEM images and selected randomly (**Figure 3**). The average diameter of the peanut shell-derived nano biochar was 21.86 ± 1.44 nm, and it was displayed as well-separated spherical nanoparticles.

**Figure 4:**
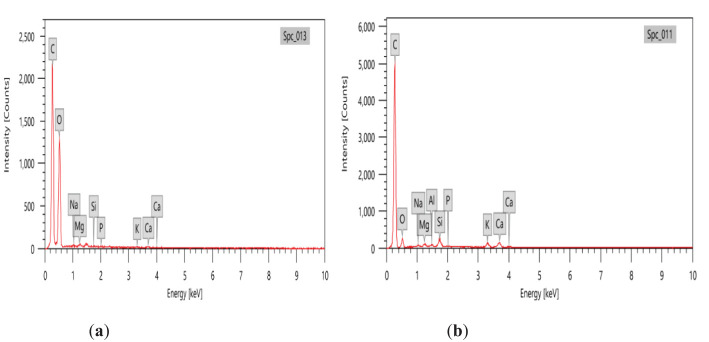
Energy-dispersive x-ray (EDX) analysis of **(A)** peanut shell and **(B)** the prepared nano biochar.

The variability in the major elements of peanut shell and its prepared nano biochar was examined by EDX (**Figure 4**). Elements such as C, O, Na, K, Ca, Mg, and Si have been detected through EDX analysis. Compared to the peanut shell, the C proportion in the prepared biochar was found to be increased, whereas O tended to decrease. The C constituent was found to be greater with an atomic weight of 86.85% in biochar prepared at 450°C, which confirmed the presence of organic matter.

### 3.2 Soil analysis

Soil physiochemical properties were significantly affected by the application of nano biochar as shown in **Table 2**. In contrast to control (no biochar) soil, the addition of 2% (w/w) and 4% (w/w) nano biochar led to a 145% and 220% increase of organic matter (OM), respectively. Moreover, nano biochar amendments significantly improved SWHC by 44.4% and 66% in B1 and B2 treatments, respectively, as compared to control soil. However, SAR decreased significantly in response to nano biochar application. The available levels of N, P, K, and Cu increased by 0.8, 10, 7, and 0.19 ppm by the application of nano biochar (B2), respectively, as compared to control soil, whereas there was no significant change in the available levels of both Zn and Mn upon nano biochar application.

**Table 2:** Physiochemical properties of control and nano biochar amendment soil.

Parameters		B0	B1	B2
**Physical properties**	Sand	94^a^ ± 4.10	86.0_b_ ± 3.32	80.0^b^ ± 2.71
	Silt	3.5^a^ ± 0.65	5.0^ab^ ± 0.70	6.0^b^ ± 0.91
	Clay	2.5^a^ ± 0.42	8.0^b^ ± 1.8	15.0^c^ ± 2.30
	SWHC	9^a^ ± 1.70	13.5^b^ ± 1.1	15.0^b^ ± 1.89
	SAR	1.9	1.7	1.6
	pH	7.97^a^ ± 0.40	7.88^a^ ± 0.20	8.10^a^ ± 0.11
	EC (dS m^−1^)	2.76^a^ ± 0.39	4.04^a^ ± 0.80	1.72^a^ ± 0.52
	OM%	0.35^a^ ± 0.12	0.86^b^ ± 0.32	1.12^b^ ± 0.24
	CaCO_3_%	2.6^a^ ± 0.61	2.9^a^ ± 0.37	3.2^a^ ± 0.41
**Available nutrients (ppm)**	N	1.50^a^ ± 0.14	2.08^ab^ ± 0.55	2.30^b^ ± 0.33
	P	18.0^a^ ± 3.78	25.0^b^ ± 2.11	28.0^b^ ± 4.10
	K	122^a^ ± 2.51	126.0^b^ ± 3.21	129.0^b^ ± 2.16
	Fe	0.73^a^ ± 0.08	1.13^b^ ± 0.32	1.21^b^ ± 0.11
	Zn	0.37^a^ ± 0.07	0.47^a^ ± 0.05	0.46^a^ ± 0.03
	Mn	0.36^a^ ± 0.03	0.39^a^ ± 0.06	0.32^a^ ± 0.01
	Cu	0.19^a^ ± 0.02	0.37^b^ ± 0.04	0.30^b^ ± 0.05

Data are presented as mean ± SD. B0: soil without biochar; B1: soil amendment with 2% nano biochar (based on soil weight), B2: soil amendment with 4% nano biochar (based on soil weight); OM: organic matter; SWHC: soil water holding capacity; SAR; sodium adsorption ratio. Different letters indicated significant difference at *p* < 0.05.

### 3.3 Effect of nano biochar and drought on growth and yield parameters

**Figure 5:**
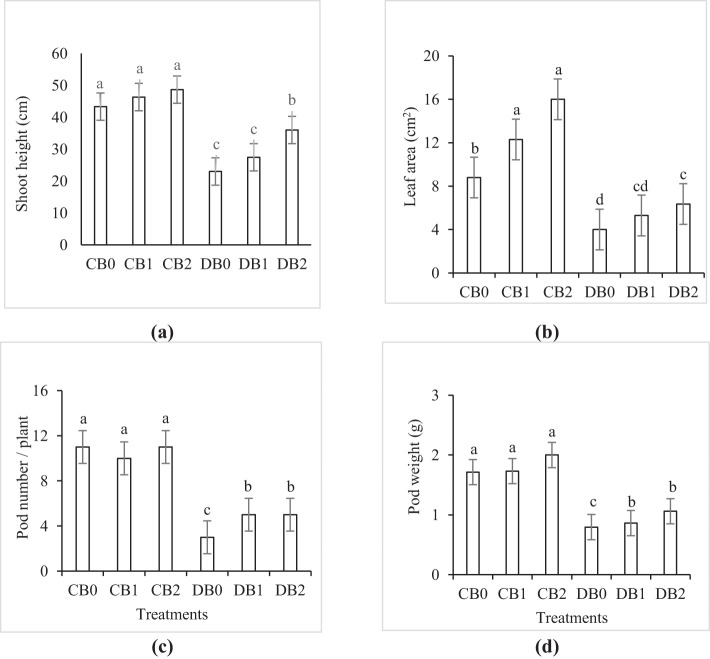
Effect of nano biochar application and drought stress on growth and yield parameters of faba bean. **(A)** Shoot height; **(B)** leaf area; **(C)** pod number/plant; **(D)** pod weight. CB0: control − zero biochar application; CB1: control + 2% nano biochar (w/w); CB2: control + 4% nano biochar (w/w); DBO: drought + zero biochar application; DB1: drought + 2% nano biochar (w/w); DB2: drought + 4% nano biochar (w/w). Different letters on bars indicated significant difference at *p* < 0.05.

Drought stress caused a significant reduction in growth and yield parameters (**Figure 5**). Compared to control plants, drought significantly reduced shoot height, leaf area, pod number/plant, and pod weight by 45%, 55%, 73%, and 116%, respectively. A significant improvement in the aforementioned parameters was noticed by 54%, 63%, 66%, and 33% compared to drought stressed plants when nano biochar was applied at a rate of 4% (w/w).

### 3.4 Effect of nano biochar and drought on photosynthetic pigments

**Figure 6:**
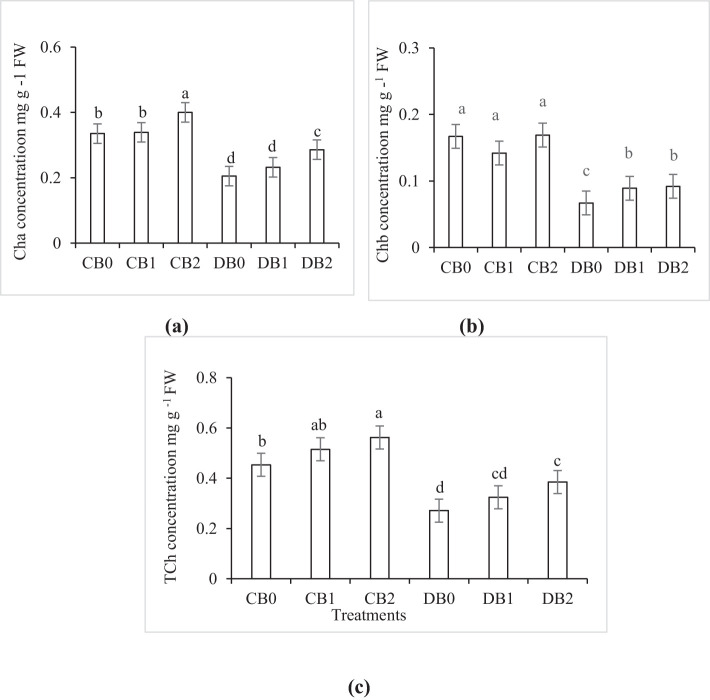
Effect of nano biochar application and drought stress on photosynthetic pigments **(A)** chlorophyll a (Cha); **(B)** chlorophyll b (Chb); and **(C)** total chlorophyll (TCh) of faba bean. CB0: control − zero biochar application, CB1: control + 2% biochar (w/w); CB2: control + 4% nano biochar (w/w); DBO: drought + zero biochar application; DB1: drought + 2% nano biochar (w/w); DB2: drought + 4% nano biochar 4% (w/w). Different letters on bars indicated significant difference at *p* < 0.05.

The effect of drought and nano biochar levels on Cha, Chb, and TCh is presented in **Figure 6**. A significant decrease in TCh content by 40% was observed under drought stress compared to control treatment. Nano biochar application at a rate of 4% (w/w) substantially improved the TCh content by 42% in case of DB2 treatment compared to DB0 treatment. The chlorophyll stability index (CSI) significantly increased from 60% in DB0 to 80% in DB2.

### 3.5 Effect of nano biochar and drought on protein content

**Figure 7:**
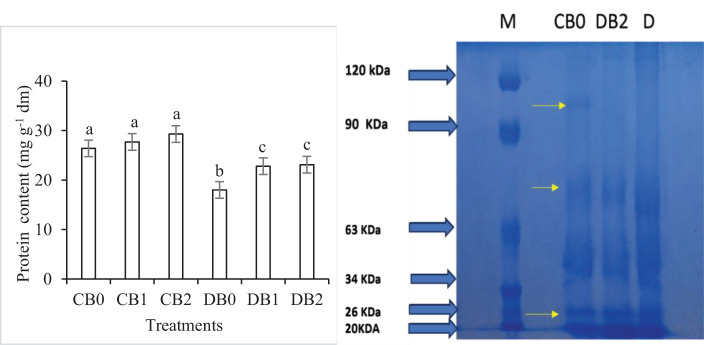
Effect of nano biochar application and drought stress on protein level and protein pattern of faba leaves. **(A)** Total protein; **(B)** Protein pattern; M: Marker; CB0: control − zero biochar application; DBO: drought + zero biochar application; DB2: drought + 4% nano biochar (w/w). Different letters on bars indicated significant difference at *p* < 0.05.

Drought stress significantly reduced protein content in faba leaves, whereas the nano biochar amendment caused a significant increase in protein content (**Figure 7**). The effect of drought and nano biochar application on polypeptide pattern of faba leaves is presented in Supplementary Figure S2. A polypeptide band with a molecular mass of 96 kDa completely disappeared in drought stressed leaves. In addition, the intensity of three bands, with a molecular mass of 72, 26, and 20 kDa, decreased in drought stressed leaves in comparison to control leaves.

### 3.6 Effect of nano biochar and drought on nutrients in leaves

**Figure 8:**
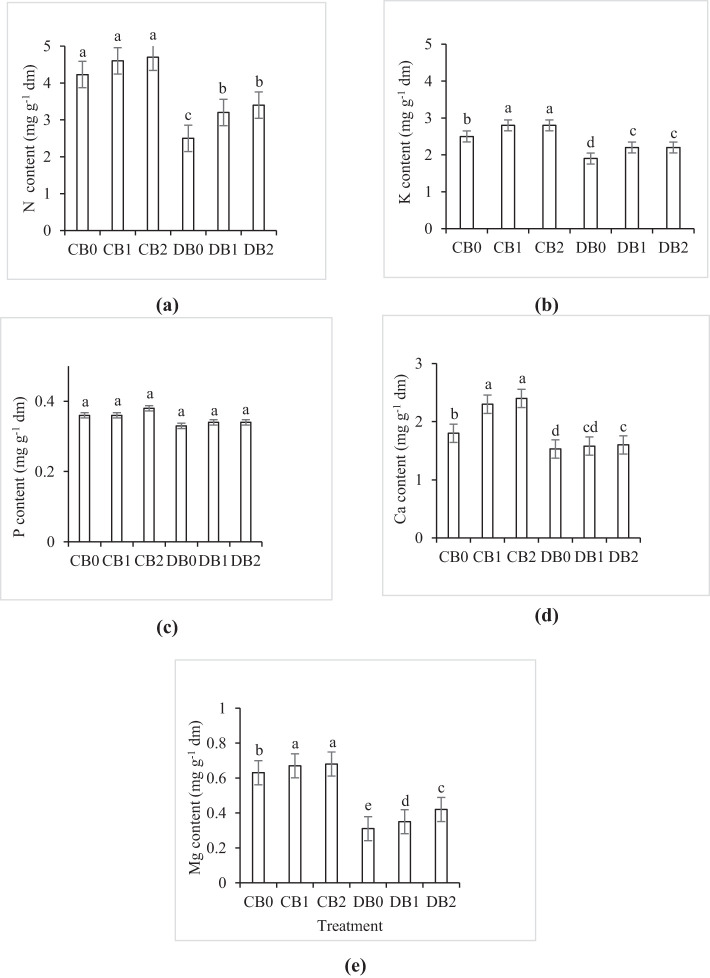
Effect of nano biochar application and drought stress on leaf elemental analysis of faba bean. **(A)** N; **(B)** K; **(C)** P; **(D)** Ca, and **(E)** Mg CB0: control − zero biochar application; CB1: control + 2% nano biochar (w/w); CB2: control + 4% nano biochar (w/w); DBO: drought + zero biochar application; DB1: drought + 2% nano biochar (w/w); DB2: drought + 4% nano biochar (w/w). Different letters on bars indicated significant difference at *p* < 0.05.

The effect of drought and nano biochar level on faba leaves’ nutrient content is shown in Supplementary Figure S3. Drought stress negatively affected the nutrient content of faba leaves. However, nano biochar amendment mitigates the negative effect of drought stress on faba leaves’ nutrient content (**Figure 8**). Maximum N (4.7), K (2.8), Ca (2.4), and Mg (0.68) content (mg g^−1^ dm) were recorded in B2 treatment, while minimum values of 2.5, 1.9, 1.53, and 0.311 (mg g^−1^ dm), respectively, were recorded in the drought stressed plants without nano biochar application. However, P content did not exhibit any difference between different treatments.

### 3.7 Effect of nano biochar and drought on lipid peroxidation and electrolyte leakage

**Figure 9:**
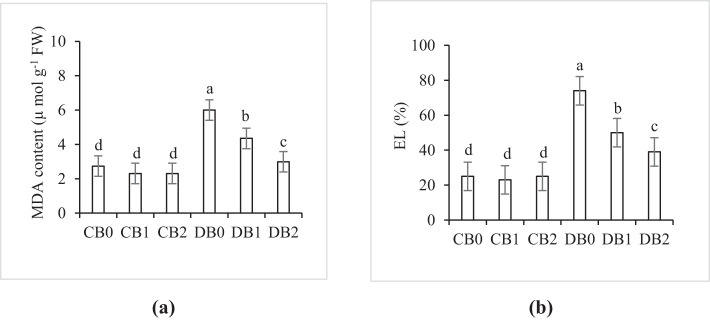
Effect of nano biochar application and drought stress on **(A)** lipid peroxidation (MDA content) and **(B)** electrolyte leakage (EL) of faba bean. CB0: control − zero biochar application, CB1: control + 2% nano biochar (w/w); CB2: control + 4% nano biochar (w/w); DBO: drought + zero biochar application; DB1: drought + 2% nano biochar (w/w); DB2: drought + 4% nano biochar (w/w). Different letters on bars indicated significant difference at *p* < 0.05.

Data presented in **Figure 9** showed that MDA, a marker of lipid peroxidation, and EL were markedly increased under drought stress. Maximum values of MDA (6 µmol g^−1^ FW) and EL (74%) were recorded in DB0 treatment, whereas minimum values were detected in CB1 and CB2. Application of nano biochar resulted in a significant decrease in MDA and EL in drought stressed faba leaves.

### 3.8 Principal component analysis

**Figure 10:**
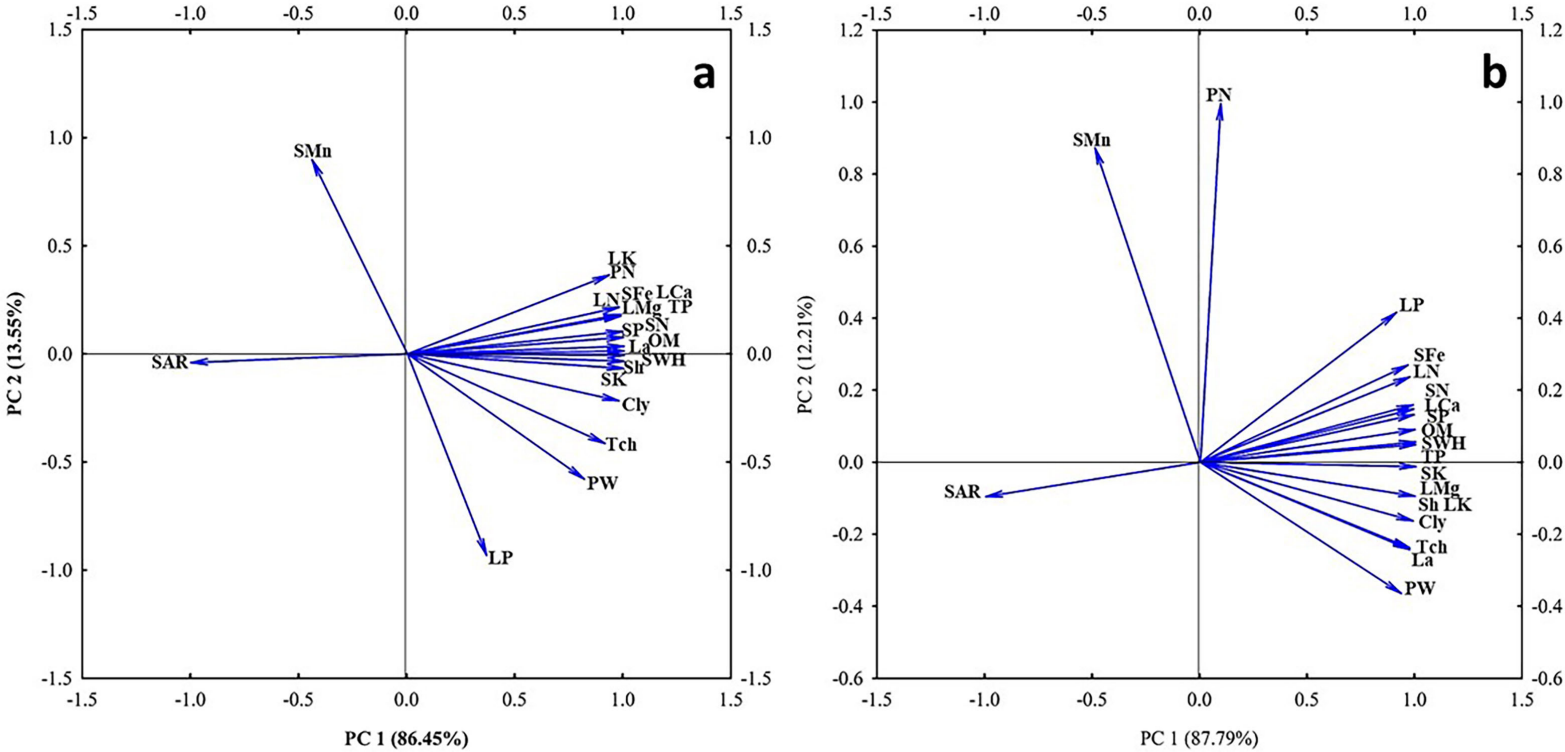
Biplot for the principal component analysis (PCA) describing correlation between the physiological response of the plants and soil parameters affected by nano biochar application. The angles between the vectors derived from the middle point of biplots exhibit positive or negative interactions of studied parameters. Biplot explains correlations between clay % (Cly), organic matter % (OM), soil water holding capacity (SWHC), sodium adsorption (SAR), soil N (SN), soil P (SP), soil K (SK), Soil Fe (SFe), soil Mn (SMn), shoot height (Sh), leaf area (La), total protein (total chlorophyll),total chlorophyll (TCh), pod weight (PW), pod number (PN), leaf N (LN), leaf P (LP), leaf Mg (LMg), and leaf Ca (LCa) under **(A)** control or **(B)** drought stress.

The PCA of correlation between the physiological response of the plants and soil parameters affected by nano biochar application is presented in **Figure 7**. The PCA showed that the first and the second dimension with eigenvalues explained the cumulative variance (100%) of the total variation in control (**Figure 10A**) and under drought stress (**Figure 10B**). The biplot graph from the PCA showed several significant correlations, including a negative correlation between SAR and the measured physiological parameters.

**Figure 11:**
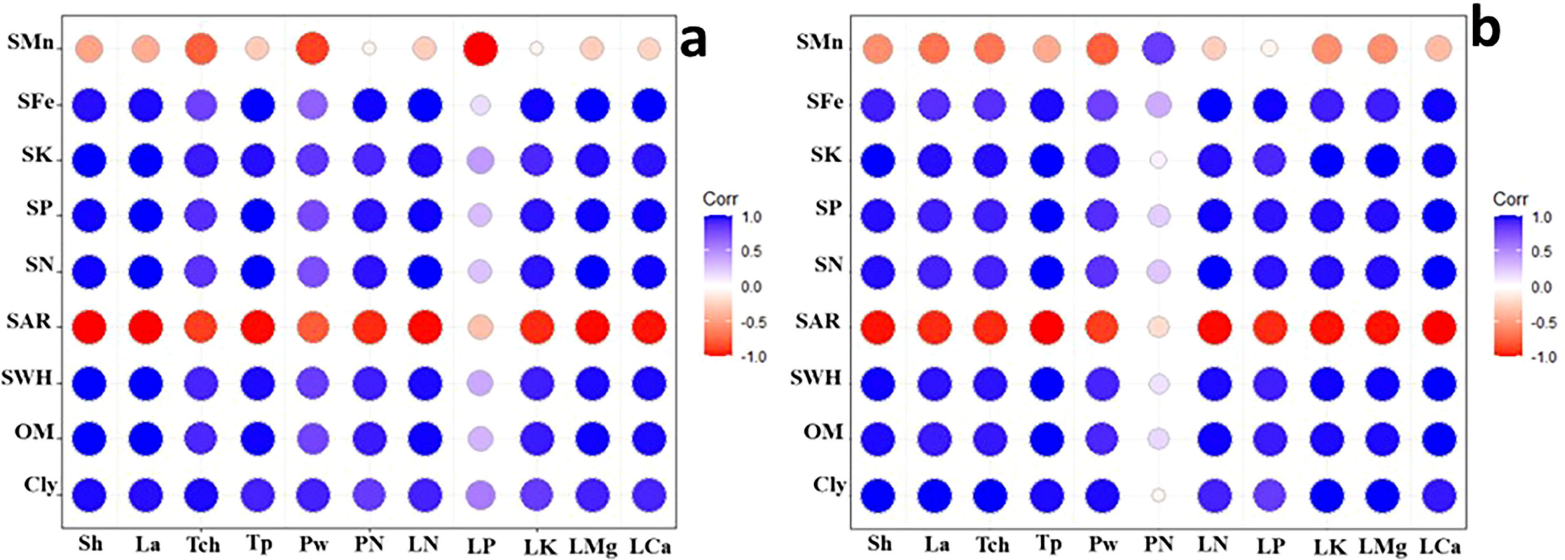
Correlogram based on the correlation coefficients between the physiological response of the plants and soil parameters affected by nano biochar application, under **(A)** control or **(B)** drought stress. Red color with −1 indicates a strong negative correlation, whereas white color with 0 means that there is no association between the two variables; however, blue color with 1 indicates a strong positive correlation. Correlation coefficients are proportional to color intensity.

Correlograms based on the correlation coefficients between the physiological response of the plants and soil parameters affected by nano biochar application under control or drought stress are shown in **Figures 11A, B**, respectively. It is apparent from the correlograms that the clay % (Cly), organic matter % (OM), and SWHC are strongly positively correlated with shoot height (Sh), leaf area (La), total protein (total chlorophyll), total chlorophyll (TCh), pod weight (PW), leaf N (LN), leaf P (LP), leaf Mg (LMg), leaf K (LK), and leaf Ca (LCa).

### 3.9 Factorial analysis

**Figure 12:**
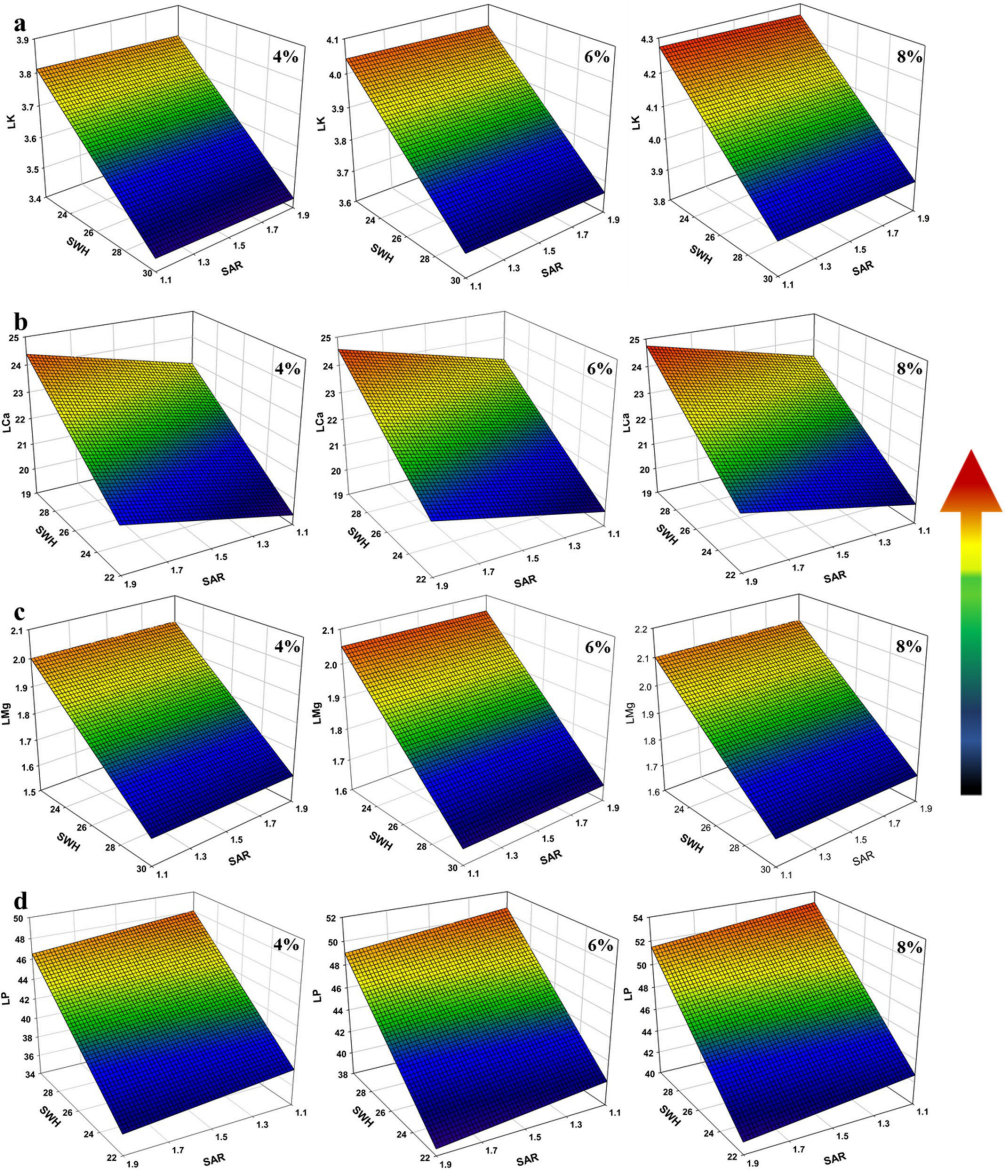
Response surface plot of **(A)** leaf K, **(B)** leaf Ca, **(C)** leaf Mg, and **(D)** leaf P as a function of soil water holding capacity (SWH) and sodium adsorption ratio (SAR) under measured (4%) and estimated (6% and 8%) nano biochar application.

**Figure 12** shows the three-dimensional response surface plot of K, Ca, Mg, and P affected by SWHC and SAR under measured (4%) and estimated (6% and 8%) nano biochar application. Increasing SWHC and decreasing SAR result in increasing the K, Ca, Mg, and P content to the maximum level.

## 4 Discussion

Peanut shell is composed of cellulose, hemicellulose, and lignin, and carbon, hydrogen, oxygen and nitrogen elements are their main contents. Generally, the surface of biochar contains plenty of oxygen-containing functional groups such as (–COOH), (–OH), (–COH), (–CONH), and lactonic groups, in addition to nitrogen-containing ones including (–NH_2_) (Yaashikaa et al., 2020). In the current study, the obtained EC value of the prepared nano biochar (3.40 dS m^−1^) is in agreement with previously stated values of Xu et al (Xu et al., 2015). The EC value is mostly related to the presence of K^+^, Ca^2+^, and Mg^2+^ (Usman et al., 2015). The obtained pH value of the prepared biochar was 7.4, even though synthesized biochar is mostly alkaline in nature, and variation greatly relies on the feedstock type and pyrolysis conditions (Novak et al., 2009). As shown in the study of Zhang et al (Zhang et al., 2017), low temperatures (<500°C) had a larger impact on biochar pH than high temperatures. The values of the bulk and tapped density for peanut shell were less dense than the prepared biochar, a finding that is in agreement with Wang et al (Wang et al., 2019).

The analysis of the FTIR spectrum of the peanut shell sample shows a broad band at 3,448 cm^−1^, which corresponds to the mixed stretching vibration absorption bands of alcoholic (–OH) and amino (–NH) groups (Dolatkhah et al., 2019; Sattar et al., 2019). The intensity of this peak decreased and showed a considerable shift to 3,400 cm^−1^ in the spectrum of the nano biochar, indicating that the conversion of the shell to nano biochar decreased the quantity of hydroxyl groups (Yuan and Xu, 2011). The aliphatic C–H stretching mode appeared at 2,923 cm^−1^ in both spectra and can be nominated to –CH_2_ stretching. The peaks at wavenumber (ʋ) 1,738 and 1,643 cm^−1^ in the spectrum of peanut shell were ascribed to the asymmetric and symmetric stretching of the carboxyl group (COOH) and/or the –COO stretching of amino acids. These two peaks disappeared in the spectrum of the prepared biochar, suggesting the decrease of –COO- content and confirming ignition loss of –COO- under oxygen environment deficiency and a pyrolysis temperature of 450°C. Moreover, thermal cracking occurs and produces volatile matter that improves the amount of aromatic surface functional groups through dehydrogenation of carbohydrates followed by a condensation process (Niazi et al., 2018). Moreover, the peak at 1,030 cm^−1^ in the peanut shell spectrum representing the vibration of the carboxylic group also disappeared in the biochar spectrum. The peak observed at ca. 1,550 cm^−1^ in both spectra may be attributed to the ether group (Dolatkhah et al., 2019), while the peak at 1,457 cm^−1^ corresponds to aromatic C=C and/or C=C–C vibrations or may be indicative of lignin and aromatic carbon. The peak at 1,426 cm^−1^ in case of peanut shell corresponds to the in-plane bending of carbonyl (–COH), which shifted to 1,430 cm^−1^ in its biochar (Yuan and Xu, 2011). The observed peaks ranging from 1,200 to 1,150 cm^−1^ may correspond to the C–O stretching of alcohols, carboxylic acids, esters, and ethers. The spectrum of peanut shell showed a peak at 1,154 cm^−1^ that may be ascribed to C–O–C symmetric stretching in ester groups of hemicellulose and cellulose. This peak slightly shifted to 1,157 cm^−1^ in the biochar spectrum. The C–O–C symmetric stretching in aliphatic groups of peanut shell appeared at 1,032 cm^−1^ (Jiang et al., 2022). The peak that appeared at 754 cm^−1^ is ascribed to the aromatic C–H out-of-plane stretching (Sattar et al., 2019).

The present study revealed that nano biochar application significantly improved soil physiochemical characteristics. These findings are in line with previous studies (Roberts et al., 2010; Rollon et al., 2020), which showed that applying biochar to agricultural land can improve the chemical and physical properties of the soil, working as an enhancer. SWHC is a primary indicator for soil quality, plant productivity, and microbial activity. The current study seems to be consistent with other studies of Basso et al. (2013) and Wang et al (Wang et al., 2019), which found that biochar amendment resulted in a significant increase in SWHC. A possible explanation for this finding is the polar interaction between OH and C–O–H functional groups on the surface of biochar and water (Lebrun et al., 2022). Furthermore, biochar might indirectly improve water retention through the addition of organic matter (Głąb et al., 2016; Jačka et al., 2018). The particle size of biochar has a vital role in regulating the storage water in soils because it has a great effect on both intrapores and interpores (Liu et al., 2017). When the biochar is added to the soil, the intrapores can hold more water and enhance water retention. Soil pore size distribution can be altered by adding biochar to soils, which affects both the movement and storage of water. The particles of the biochar can fill the big pore spaces that exist in rough grained soils, thus enhancing retention and decreasing the water flow rate (Blanco-Canqui, 2017). In this work, the average diameter of the synthesized nano biochar particles was 21.86 nm. As reported in the literature, a small biochar particle size greatly affects soil water retention compared to a large particle size, because it has a large surface area that can fit easily into the sandy soil’s large pores. Moreover, it can greatly increase meso-porosity even at lower application rates (Edeh and Mašek, 2022). In contrast to these studies, Hardie et al. (2014) and (Jeffery et al. (2015) demonstrated that biochar amendment did not show any significant improvement in soil water retention.

In this work, drought negatively affected the growth, yield, and protein content of faba bean. These results are consistent with many previous studies, which recorded that drought has a devastating effect on plant growth and yield (Farooq et al., 2008; Osakabe et al., 2014; Fahad et al., 2017). However, the application of nano biochar under drought stress resulted in a significant increase in growth, yield, and protein content of faba bean plants. The present findings seem to be consistent with other research that found that applying biochar increased the growth and yield of a number of drought-stressed plants such as tomato (Akhtar et al., 2014), maize (Haider et al., 2015), and okra (Batool et al., 2015). In addition, Sammar Raza (Raza et al., 2023) reported that application of nano biochar improved wheat growth under drought stress. This beneficial role of biochar in alleviating the harmful effects of drought stress in faba bean plants may result from its ability to increase SWHC and organic matter and decrease SAR. Abed Hussein et al. (2022) stated that the addition of nano biochar to the soil resulted in a significant increase in the level of organic matter. In the current study, there is a strong positive correlation that appeared in the correlogram between organic matter % (OM), SWHC, and physiological responses of faba plants (**Figure 8**). These findings further support the idea of Foster et al. (2016) who stated that the vital role of biochar in improving SWHC is a key factor in enhancing plant growth and yield during drought stress. In addition, Govindasamy et al. (2023) reported that a management’s practice effect on SWHC, whether positive or negative, could directly indicate its possible impact on plant yield.

The application of nano biochar resulted in a significant increase in the levels of available N, P, K, Zn, and Cu. The present findings seem to be consistent with other research, which found that the addition of biochar to soil may improve its nutrient availability (Diamadopoulos, 2019; Edeh and Mašek, 2022). A possible explanation for this enhanced nutrient availability might be the large specific surface and small particle size of the nano biochar that enables it to couple easily with nutrients to become a high-efficiency fertilizer (Guo et al., 2013). Moreover, the low BD of the prepared nano biochar allows it to keep the nutrients and water content, thus minimizing the hardening of soil [application of nano biochar increased SWHC (by 66%), N (by 53%), P (by 55%), and K (by 7%)]. The observed surface area of the prepared nano biochar in the current study (208.2 m^2^ g^−1^) represented the impact of the utilized pyrolysis temperature on carbonization. During pyrolysis, the release of volatile matter produced from celluloses and hemicelluloses can enhance the formation of vascular bundle structure in the biochar and thus expand the specific surface area of the biochar. The release of O and H bearing functional groups of phenolic (–OH), alkyl (–CH2), aromatic (–CO), and ester C=O groups could result in the high surface area value, which forms a high sorption capacity to keep soil moisture and nutrients, provides a refuge for beneficial soil microorganisms, minimizes the hardening of soil, and enhances the availability of nutrients (Liang et al., 2006; Agegnehu et al., 2015). In line with this, nano biochar amendment boosted the mineral content of N, K, Ca, and Mg in faba bean leaves under drought stress. In contrast, Brtnicky et al. (2021) and Zhao et al. (2019) found that biochar application may decrease nutrient availability because of its high sorption capacity.

In the current study, the total chlorophyll content of faba bean decreased significantly under drought stress. Similarly, Kiani et al. (2020) reported that water stress disturbs photosynthetic apparatus and decreases the synthesis of photosynthetic pigments. One of the defense strategies the plant uses to lower the quantity of energy received by the leaf during a drought is the loss of photosynthetic pigments (Liu et al., 2017). The significant increase in total chlorophyll content of faba bean in response to nano biochar application of the current study corroborates the ideas of (Azadifar et al. (2023) who stated that nano biochar amendment increased chlorophyll content in broccoli under deficit irrigation. This could be associated with the improvement in mineral content resulting from biochar application (Abideen et al., 2020).

Drought leads to the excessive formation of ROS, which has high affinity to react with nucleic acids, lipids, and proteins, leading to malfunctioning of these macromolecules and accelerating the aging process (Kocsy et al., 2013; Rizwan et al., 2015). In the current study, drought stress elevated MDA and EL %, whereas nano biochar amendment resulted in a significant decrease in the aforementioned parameters. These results, consistent with those of Lyu et al. (2016) and Gharred et al (Gharred et al., 2022), suggest that biochar application under drought stress led to a reduction in the level of MDA. Recently, Rasheed et al. (2024) reported that application of nano biochar enhanced membrane stability and reduced EL in spinach under conditions of drought and salt stress. This could be attributed to the improvement in the WHC and nutrient availability of the soil, which helps in drought amelioration.

The use of nano biochar in soil amelioration depends on several factors, such as the method of preparation and modification of biochar (Yang et al., 2019), type of feedstock (Wijitkosum, 2022), and rate of application (Yeboah et al., 2016). Applying too much biochar can disturb the nutrient availability in soil (Jiang et al., 2022). Surprisingly, factorial analysis of this study indicates that no significant differences were found in the levels of N, K, Mg, and Ca in faba bean plants when biochar was applied at rates of 6% and 8% (expected rates) compared to 4% (measured), as demonstrated in the three-dimensional response surface plot (**Figure 9**). Hence, the application of biochar as a soil improver is less expensive and more environmentally sustainable.

## 5 Conclusion

The current study showed that drought caused a significant reduction in growth and yield parameters of faba bean. Cost-effective and eco-friendly nano biochar is produced from peanut shells as a feedstock. The application of the synthesized nano biochar resulted in a significant increase in SWHC, organic matter content, and nutrient levels, which are primary indicators of biochar’s beneficial effects on soil. These improvements were reflected in growth, yield, and a decrease in OS, as indicated by the reduced levels of lipid peroxidation and EL in faba beans under drought stress in the presence of biochar. The principal component and factorial analysis of the present study showed the relationship between the physiological response of faba bean plants and soil parameters affected by the application of nano biochar. This study sheds light on a sustainable way of disposing peanut shells as agricultural waste, which copes with the sustainable development goals declared by the United Nations (Farooq et al., 2008; Ali et al., 2017; Abid et al., 2020; Kumar et al., 2020).

## Data Availability

The original contributions presented in the study are included in the article/supplementary material. Further inquiries can be directed to the corresponding author.
